# Human Milk Antioxidative Modifications in Mastitis: Further Beneficial Effects of Cranberry Supplementation

**DOI:** 10.3390/antiox11010051

**Published:** 2021-12-27

**Authors:** Victoria Valls-Bellés, Cristina Abad, María Teresa Hernández-Aguilar, Amalia Nacher, Carlos Guerrero, Pablo Baliño, Francisco J. Romero, María Muriach

**Affiliations:** 1 Unitat Predepartamental de Medicina, Facultat de Ciencies de la Salud, Universitat Jaume I, 12071 Castellon de la Plana, Spain; vallsv@uji.es (V.V.-B.); cristina.abad@cofcastellon.org (C.A.); lactancia_peset@gva.es (M.T.H.-A.); nacher_ama@gva.es (A.N.); cguerrer@uji.es (C.G.); balino@uji.es (P.B.); 2 Hospital General de Requena, Conselleria de Sanitat, Generalitat Valenciana, 46340 Requena, Spain

**Keywords:** mastitis, human milk, oxidative status, cranberry

## Abstract

Mastitis is the inflammation of one or several mammal lobes which can be accompanied by a mammary gland infection, and is the leading cause of undesired early weaning in humans. However, little information exists regarding the changes that this disease may induce in the biochemical composition of human milk, especially in terms of oxidative status. Given that newborns are subject to a significant increase in total ROS burden in their transition to neonatal life and that their antioxidant defense system is not completely developed, the aim of this study was to evaluate antioxidant defense (glutathione peroxidase (GPx), reduced glutathione (GSH), total polyphenol content (TPP), and total antioxidant capacity (TAC)) in milk samples from mothers suffering from mastitis and controls. We also measured the oxidative damage to lipids (malondyaldehyde (MDA)) and proteins (carbonyl group content (CGC)) in these samples. Finally, we tested whether dietary supplementation with cranberries (a product rich in antioxidants) in these breastfeeding mothers during 21 days could improve the oxidative status of milk. GPx activity, TPP, and TAC were increased in milk samples from mastitis-affected women, providing a protective mechanism to the newborn drinking mastitis milk. MDA concentrations were diminished in the mastitis group, confirming this proposal. Some oxidative damage might occur in the mammary gland since the CGC was increased in mastitis milk. Cranberries supplementation seems to strengthen the antioxidant system, further improving the antioxidative state of milk.

## 1. Introduction

Human milk is characterized by a huge variability in its composition, as it includes nutrients as well as bioactive compounds and a vast array of microbes known as the human milk microbiota. As the neonatal immune system develops, this milk variability and composition provides the infant with well-balanced nutrition and protection against potential infectious pathogens [1,2]. The World Health Organization (WHO) recommends that infants be exclusively breastfed for the first six months of life for optimal growth, development, and health, and that breastfeeding continues to be an important part of the diet until the infant is at least two years old [3].

With regard to bioactive compounds, the antioxidant content of breast milk has been the subject of a number of studies, confirming the presence of different components that have been reported to modulate the effects of oxidative stress [4,5,6,7,8,9,10]. Birth represents a significant oxidative challenge because it involves the transition from the relatively hypoxic intrauterine conditions to the oxygen-rich extrauterine environment. Thus, newborns are subject to a significant increase in total ROS burden in their transition to neonatal life [11,12]. 

This situation is especially relevant in premature newborns due to their undeveloped antioxidant defense system [13,14]. Moreover, increasing evidence also indicates that some pathological conditions appearing in adulthood (such as type 2 diabetes, hypertension, obesity, and others) associated with oxidative stress may have their origin in the fetal or neonatal period of life [15,16,17]. 

Mastitis is the inflammation of one or several mammal lobes which can be accompanied by a mammary gland infection, and is the leading cause of unwelcome early human weaning. The primary origin of mastitis is milk stasis [18], and its incidence varies from 2% to 33% according to different authors, being more frequent during the early postpartum weeks. Infective mastitis is usually associated with the growth of certain species of staphylococci, streptococci, and/or corynebacteriae in the milk [19,20]. Treatment traditionally consists of antibiotic therapy, analgesic medication, and proper milk removal [21]. However, the WHO has highlighted significant concerns regarding the adverse side effects of antibiotics due to the emergence of antibiotic-resistant strains of microorganisms. In this context, more recently specific probiotic bacteria have been postulated to prevent mastitis due to their anti-inflammatory and immunomodulatory properties [22]. 

In fact, almost all of our knowledge regarding the biology of human mastitis is extrapolated from bovine or goat studies [23]. The group of Gelasakis and colleagues demonstrated lower fat and lactose content in milk from goats with subclinical mastitis, together with a mild increase in protein content [24]. Similar results were obtained by Sun et al. in bovine mastitis [25]. Furthermore, it has been reported that subclinical mastitis in goats upregulates nitric oxide-derived oxidative stress and reduces milk antioxidant properties [26]. However, it is noteworthy that two recent studies also described the effect of lactational mastitis on the macronutrient content of human breast milk [27,28] in accordance with the above-mentioned results reported in bovine and goats. In addition, Perez et al. reported that mastitis modifies the biogenic amine profile of human milk [29]. Interestingly, it has been described that human mastitis milk has the same anti-inflammatory components and characteristics of normal milk, with elevations in selected components/activities (not including antioxidant activity in terms of spontaneous cytochrome c reducing activity) that may help protect the nursing infant from developing clinical illness due to feeding on mastitis milk [30]. This work aims to study oxidative status in milk from mothers with mastitis. Moreover, we extended the present investigation to study whether dietary supplementation with cranberries (*Vaccinium* sp.) a product known to be rich in antioxidants, could improve oxidative status in human milk [31,32].

## 2. Materials and Methods

### 2.1. Experimental Design

In total, 60 non-smoking lactating mothers who had delivered to term and breastfed their babies starting from the first day postpartum were included in the study. The study was approved by the Research Ethics Committees from both, Hospital General Universitario from Castellón (4/2015) and Hospital Universitario Dr. Peset from Valencia (23/16). All the participants were fully informed of the procedure and gave their written consent to participate. They were also allowed to withdraw from the study at will. Of these mothers, 30 were healthy lactating mothers and the other 30 referred to symptoms of mastitis which included breast swelling and redness together with pain or a burning sensation continuously or while breast-feeding. No fever or abscess were present in any case. The microbiological culture confirmed the bacterial infection in all cases, and therefore all subjects in the mastitis group received antibiotic treatment during the study. A total of 15 subjects from each group accepted dietary supplementation with cranberries (20 g/day) for 21 days. Thus, subjects were finally assigned to 4 groups (*n* = 15): control (C), control + cranberries (C + C), mastitis (M), and mastitis + cranberries (M + C).

Vaccinium berry fruits are widely known for their health benefits. These particular, berry species present high concentrations of antioxidants, including phenolic compounds, and the presence of specific, particularly potent polyphenolic compounds [31,32].

Two samples of mature milk were obtained from each mother, with one taken at the beginning of the study and another after 21 days. Prior to sampling, all donors gave informed consent regarding participation in the study and completed a questionnaire on nutritional habits, with the purpose of confirming the homogeneity of the population. Furthermore, the demographic characteristics of the subjects were recorded, and dietary intake was assessed using 24 h recall. This dietary assessment was performed over 3 days (1 holiday and 2 working days). The food ingested on the different days was processed by the “Alimentador” software of the Sociedad Española de Dietética y Ciencias de la Alimentación (SEDCA).

### 2.2. Collection of Samples

Approximately 10 mL of mature milk was collected prior to feeding of the nursing infant using an electric breast pump fitted with a vacuum regulator (MEDELA) connected to polypropylene containers in which the samples were collected directly. After milk extraction, the samples were immediately transported to liquid N_2_ storage in dark conditions to avoid oxidative processes. The samples were subsequently divided into 4 aliquots and frozen at −80 °C.

### 2.3. Methods

The oxidative status of human milk samples was assessed in this study. For this purpose, on one hand the total antioxidant capacity (TAC), glutathione peroxidase (GPx) activity, reduced glutathione (GSH) concentration, and total polyphenol content (TPP) values were measured. On the other hand, oxidative damage to macromolecules was assessed by measuring malondialdehyde (MDA) as a lipid peroxidation product and carbonyl group content (CGC) as a marker of oxidative damage to proteins.

### 2.4. TAC Determination

The ABTS assay to determine TAC was modeled after the method proposed by Miller and Rice Evans [33] and modified by Re et al. [34]. Briefly, the assay is based on the oxidation of ABTS by potassium persulphate to form the radical monocation ABTS^•+^, which is reduced in the presence of hydrogen-donating antioxidants. The reagent ABTS^•+^ was generated by exposing a 7 mM solution of ABTS to a solution of 2.45 mM potassium persulphate at a 1:1 ratio. The assay included 2.970 mL of ABTS^•+^ and 30 μL of plasma. Antioxidant activity was determined by measuring the decolorization of the ABTS^•+^ (reduction of the radical cation) through the absorbance at 734 nm.

### 2.5. Assay of GPx Activity

Glutathione peroxidase activity, which catalyzes the oxidation by H_2_O_2_ of GSH to its disulfide (GSSG), was assayed spectrophotometrically as reported by Lawrence et al. [35] by monitoring the oxidation of NADPH at 340 nm. The reaction mixture consisted of 240 mU/mL of GSH disulfide reductase, 1 mM GSH, and 0.15 mM NADPH in 0.1 M potassium phosphate buffer at pH 7.0 containing 1 mM EDTA and 1 mM sodium azide. A 50 μL sample was added to this mixture and allowed to equilibrate at 37 °C for 3 min. The reaction was started by the addition of hydrogen peroxide to adjust the final volume of the assay mixture to 1 mL.

### 2.6. Assay of GSH Concentration

GSH concentration was quantified following the method of Reed et al. [36] based on the reaction of iodoacetic acid with the thiol groups followed by a chromophore derivatization of the amino groups with Sanger reactant (1-fluoro-2,4-dinitrobencene), giving rise to derivatives which are quickly separated by means of high-performance liquid chromatography (HPLC).

### 2.7. TPP Determination

To measure the total content of polyphenols in breast milk, the method of Folin Ciocalteu was used [37]. This method is based on the oxidation of polyphenols with the Folin reagent. When milk is mixed with the reagent, the formation of a blue complex is achieved, for which absorbance is measured at 750 nm. Briefly, milk samples were centrifuged at 12,000× *g* for 5 min. Then, 500 µL of supernatant was taken and 100 µL of 1.32 M metaphosphoric acid was added for protein precipitation. The mixture was centrifuged at 2700 rpm for 3 min, and the supernatant was collected. Then, 20 µL of the supernatant, 80 µL of distilled water, and 500 µL of reagent were mixed. Subsequently, 400 µL of 7.5% sodium carbonate was added and measured at 750 nm, using gallic acid as standard.

### 2.8. Assay of MDA Concentration

MDA concentration was measured by liquid chromatography according to a modification [38] of the original method of Richard et al. [39]. Briefly, 0.1 mL of sample (or standard solutions prepared daily from 1,1,3,3-tetramethoxypropane) and 0.75 mL of working solution (thiobarbituric acid 0.37% and perchloric acid 6.4%; 2:1, *v*/*v*) were mixed and heated to 95 °C for 1 h. After cooling for 10 min in an ice water bath, the flocculent precipitate was removed by centrifugation at 3200× *g* for 10 min. The supernatant was neutralized and filtered (0.22 µm) prior to injection on an ODS 5 µm column (250 × 4.6 mm). The mobile phase consisted of 50 mM phosphate buffer (pH 6.0): methanol (58:42, *v*/*v*). Isocratic separation was performed with 1.0 mL/min flow and detection at 532 nm.

### 2.9. Quantification of CGC

Carbonyl groups were determined to evaluate protein oxidation in milk samples. The CGC released during incubation with 2,4-dinitrophenylhydrazine was measured using the method reported by Levine et al. [40]. Briefly, the samples were centrifuged at 13,000× *g* for 10 min. Then, 20 mL of brain homogenate was placed in a 1.5 mL Eppendorf tube, and 400 mL of 10 mM 2,4 dinitrophenylhydrazine/2.5 M hydrochloric acid (HCl) and 400 mL of 2.5 M HCl were added. This mixture was incubated for 1 h at room temperature. Protein precipitation was performed using 1 mL of 100% TCA, and the mixture was washed twice with ethanol/ethyl acetate (1/1, *v*/*v*) and centrifuged at 12,600× *g* for 3 min. Finally, 1.5 mL of 6 N guanidine (pH 2.3) was added and the samples were incubated in a 37 °C water bath for 30 min and centrifuged at 12,600× *g* for 3 min. The carbonyl content was calculated from peak absorption (373 nm) using an absorption coefficient of 22,000 M^−1^ cm^−1^ and was expressed as nmol/mg protein.

### 2.10. Protein Concentration Measurement

Protein content was determined using Bradford method. 

### 2.11. Statistical Analysis

The statistical analyses were performed using SPSS 24 (IBM SPSS, Chicago, IL, USA). The Kolmogorov test was used to check the normal distribution of each population (*p* > 0.100) to analyze the data based on parametric analysis of variance (ANOVA). 

Generally, multifactorial ANOVA is applied to analyze the differences between data groups (in this case for example according to the influence of the cranberry supplement and considering time as a second factor). However, given that the “time” factor includes only two measurements, we finally decided to use 1-way ANOVA using the factor of “treatment”, with the studied variable being the difference in the results for each parameter measured at the beginning and the end of the study. Statistical significance was considered at *p* < 0.05. Significant differences in each sampling group were assessed using the least significant difference (LSD) procedure. 

## 3. Results

The obtained anthropometric measurements and dietetic assessment results are listed in Table 1. There were no differences in age, weight, height, and BMI among the different groups in the study. Significant differences were not found in terms of macronutrient and micronutrient intakes among the four groups.

**Figure 1:**
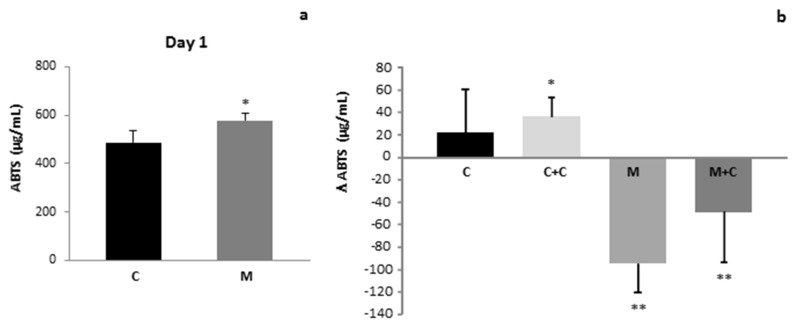
(**a**) Total antioxidant capacity of human milk of Control and Mastitis groups (*p* < 0.05 vs. C). (**b**) Variation of total antioxidant capacity of human milk along the study of Control, Control + Cranberries, Mastitis and Mastitis + Cranberries groups. Control (C), Control + Cranberries (C + C), Mastitis (M) and Mastitis + Cranberries (M + C). * *p* < 0.05 vs. M and M + C, ** *p* < 0.05 vs. rest of groups.

Regarding the oxidative status of human milk, TAC was higher at the beginning of the study in milk from the mastitis group than in control milk. Twenty-one days later the values for this parameter were similar to those of the control. When the TAC evolution in every group throughout the study was analyzed, a significant decrease in TAC in milk samples from the mastitis group was observed when compared to the control group (Figure 1b). Furthermore, Figure 1b shows that cranberry intake significantly increased TAC in control and mastitis milk samples.

**Figure 2:**
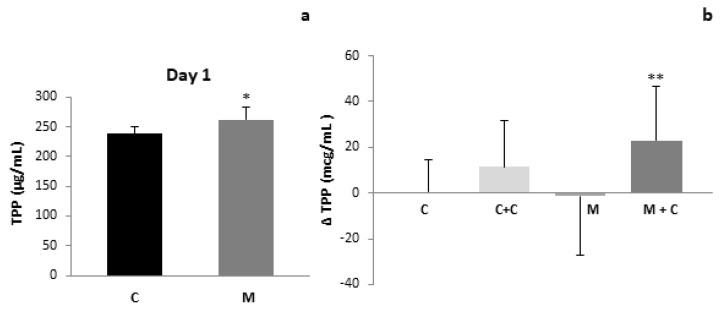
(**a**) Total polyphenol content of human milk of Control and Mastitis groups (*p* < 0.05 vs. C). (**b**) Variation of total polyphenol content of human milk along the study of Control, Control + Cranberries, Mastitis and Mastitis + Cranberries groups. Control (C), Control + Cranberries (C + C), Mastitis (M) and Mastitis + Cranberries (M + C). * *p* < 0.05 vs. C, ** *p* < 0.05 vs. C and M.

An increase in TPP content in mastitis samples at the beginning of the study was observed (Figure 2a). Similar to what occurs to TAC, TPP content normalized after 21 days. Cranberry intake increased TPP content in milk samples during the study, although this was significant only in the mastitis group (Figure 2b).

**Figure 3:**
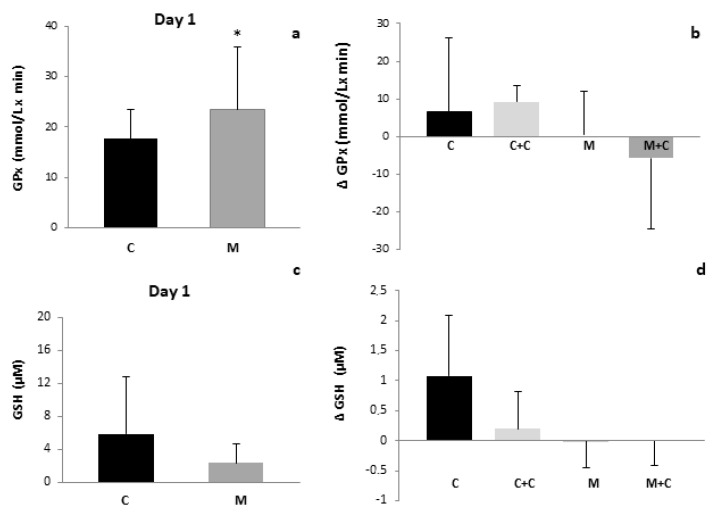
(**a**). GPx activity of human milk of Control and Mastitis groups (*p* < 0.05 vs. C). (**b**). Variation of GPx activity of human milk along the study of Control, Control + Cranberries, Mastitis and Mastitis + Cranberries groups. * *p* < 0.05 vs. C. (**c**). GSH concentration of human milk of Control and Mastitis groups (*p* < 0.05 vs. C). (**d**). Variation of GSH concentration of human milk along the study of Control, Control + Cranberries, Mastitis and Mastitis + Cranberries groups. Control (C), Control + Cranberries (C + C), Mastitis (M) and Mastitis + Cranberries (M + C). * *p* < 0.05 vs. C.

The GSH concentration of milk was not significantly affected by mastitis (Figure 3a). In contrast, GPx activity was increased in the milk of the mastitis group as compared to the control at the beginning of the study (Figure 3c), but again normalized 21 days later. No significant changes were observed in the evolution of this parameter in any group over the 21 days of the study. Cranberry intake had no effect on GSH concentrations nor GPx activity (Figure 3b,d, respectively).

**Figure 4:**
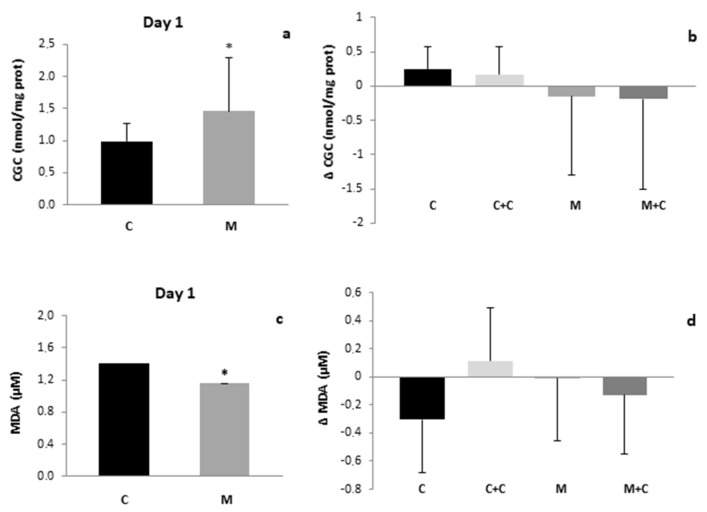
(**a**) Carbonyl groups content of human milk of Control and Mastitis groups (*p* < 0.05 vs. C). (**b**) Variation of Carbonyl groups content of human milk along the study of Control, Control + Cranberries, Mastitis and Mastitis + Cranberries groups. (**c**) MDA concentration of human milk of Control and Mastitis groups (*p* < 0.05 vs. C). (**d**) Variation of MDA concentration of human milk along the study of Control, Control + Cranberries, Mastitis and Mastitis + Cranberries groups. Control (C), Control + Cranberries (C + C), Mastitis (M) and Mastitis + Cranberries (M + C). * *p* < 0.05 vs. C.

Greater oxidative damage to proteins was observed in mastitis milk samples as compared to the control at the beginning of the study (Figure 4a). However, this difference was not statistically significant in the samples obtained after 21 days. Nor were significant differences observed in the evolution of this parameter within groups throughout the study (Figure 4b). Contrary to the findings of CGC, MDA concentration in milk was significantly lower in the mastitis group at the beginning of the study as compared to the control (Figure 4c). Similar to CGC, after 21 days MDA concentration was normalized. Figure 4d shows that MDA concentrations were not affected by cranberry intake.

## 4. Discussion

In recent decades there have been significant lifestyle changes in the human population, with a tendency towards an increased consumption of processed foods and a consequent rearrangement of dietary patterns [41,42]. The results from the present study demonstrate insufficient nutrient intake in the study population with respect to WHO recommendations [3], i.e., daily fruit and vegetables, as well as carbohydrate intakes were below those recommended by WHO, whereas fat and protein consumption was above WHO recommendations [3].

It has been widely reported that breast milk has a powerful and essential antioxidant composition, which is related to the combination of both exogenous and endogenous molecules including, among others, enzymes, vitamins, protein components and derivatives, oligoelements, carotenoids, and polypohenols [7,17]. Mastitis is associated with inflammatory processes and innate immune cell recruitment and activation, which in turn results in the release of proinflammatory cytokines [43]. Although leukocyte recruitment can also increase the local free radical production to unbalanced levels, thus compromising the oxidative status of milk, as has been previously described in cows, goats, and sheep [44,45,46], it is not clear whether the anti-inflammatory content of human milk in mastitis affected women, provides an extra protection against these changes [30]; these authors proposed that the increased contents of selected components in womens’ mastitis milk (e.g., TNFa or IL-1RA) might help protect the nursing infant from clinical illness due to feeding on mastitis milk [30]. 

To the best of our knowledge our study shows for the first time that total antioxidant capacity (TAC) was increased (Figure 1), along with GPx activity and TPP content in milk samples from women suffering from mastitis when compared to the control group (Figure 2 and Figure 3a,b). These results may be explained as a compensatory adaptation to oxidative stress that has previously been described in other pathological conditions, in which the antioxidative response is stimulated to reduce the oxidant content and reestablish the redox balance [47,48], or a breast-specific mechanism that increases permeability of the mammary gland for certain components. Regarding the GPx increase, it has been previously reported that lactoferrin (LF) concentration is increased in human breast milk during mastitis [27,49]. This protein is known to upregulate the expression of GPx [50] and to enhance its activity [51]. On the other hand, recent studies have demonstrated that mastitis is associated with an increase in milk selenium (Se) concentrations [27]; selenocysteine is present in the active site of GPx [52]. Therefore, the increase in milk Se concentration could certainly contribute to the enhanced GPx activity in milk from the mastitis group as compared to the control.

Polyphenols are present in human milk, of which flavonoids represent the largest subgroup [53]. Li et al. compared polyphenol content in human breast milk with that in formula [54]. However, as mentioned above, at present there are no studies addressing the effect of mastitis on human milk TPP content. 

In contrast to the findings on GPx activity and TPP content, no increase in GSH concentration was observed in milk from the mastitis group (Figure 3c,d), in agreement with former results in bovine milk [44]; although these authors reported a decrease of TAC in bovine milk. Furthermore, Dimri et al. also described a decrease in milk TAC together with lower GPx activity in buffaloes [55]. These discordances may be due to interspecies differences. 

Interestingly, 21 days after mastitis onset, values for all the parameters studied returned to normal, probably due to the effect of antibiotics. Moreover, 21 days cranberry supplementation increased TTP content in milk samples from both the control and mastitis group (Figure 2), since cranberries are fruits that are especially rich in polyphenols [56]. Other authors have previously demonstrated that dietary supplementation with foods rich in these compounds are able to increase the quantities of flavonoids in human milk [57,58]. Similar results were obtained for TAC in milk after supplementation with cranberries (Figure 1). Other studies in animal models of mastitis have also focused on the role of natural antioxidants such as vitamin E or ginsenoside Rg1 in protecting the oxidative status of milk [55,59], although no studies are available on human milk.

With regard to oxidative damage to macromolecules, there are no reports at present showing the effects of mastitis in terms of oxidative damage to proteins. We have demonstrated that mastitis induces oxidative damage to milk proteins (Figure 4). This result is consistent with the increase in mastitis milk protein content reported by Samuel and cols. [27]. In contrast to the observed oxidative damage to proteins, lipid peroxidation (MDA concentration) was diminished in milk from the mastitis group when compared to the control (Figure 4a,b). This is a surprising result, given that lipids are more sensitive to oxidative damage than proteins. In this respect, it is noteworthy that Say et al. recently evidenced that lactational mastitis is associated with lower breast milk fat [28], and therefore it is possible that the decrease in MDA concentration in the mastitis group was due, at least in part, to the diminished fat content. Furthermore, as mentioned above, recent studies have reported that mastitis increases LF concentrations [27,50]. LF sequesters Fe^3+^, preventing the formation of hydroxyl radicals via the Fenton reaction, and in turn prevents lipid peroxidation [51,60]. Therefore, the increase in LF content in milk could also contribute to the decrease in MDA concentration observed in the mastitis group (Figure 4a,b). Again, these consequences of mastitis could be specific to lactating human females, since lipid peroxidation products are increased in milk samples from rats or cows with mastitis [61,62].

Both CGC and MDA concentrations normalized at the end of the study, after antimicrobial therapy (Figure 4), which probably reduces the macronutrient content impairment described previously [27,28]. It is remarkable that cranberry supplementation had no effect on these oxidative damage parameters (Figure 4), probably because cranberries are able to attenuate the oxidative insult by increasing overall antioxidant defense. Many studies have assayed therapies with antioxidants in different animal models to prevent the effects of mastitis on the oxidative status of milk [55,59,63]. The results have been mainly successful, but some controversial results have also been reported regarding the use of several antioxidants such as vitamin E [64,65]. However, at present there are no studies reporting on the antioxidative status of milk from women with mastitis or the effects of antioxidant supplementation on this status in human milk.

In conclusion, during mastitis there is a compensatory mechanism in human milk in which antioxidant defenses are increased, providing the nursing infant with further protection while feeding on mastitis milk. In addition, the use of cranberries as supplement for antibiotic therapy seems to strengthen the antioxidant system of milk.

## Figures and Tables

**Table 1:** Anthropometric measurements and dietetic assessment (daily average).

	C	C + C	M	M + C
Age	36.5 ± 4.1	33.8 ± 3.7	33.7 ± 3.9	36.4 ± 3.3
Weight (kg)	60.6 ± 7.2	64.6 ± 8.8	66.2 ± 10.5	63.9 ± 10.5
Height (cm)	164.6 ± 4.7	165.5 ± 6.2	163.2 ± 4.8	164.6 ± 6.7
BMI	22.3 ± 2.5	23.6 ± 2.6	24.8 ± 3.9	23.6 ± 4
Kcal	1595 ± 394	1510 ± 265	1659 ± 248	1631 ± 428
Carbohydrates (g)	166.2 ± 54.5	167.8 ± 48	191.2 ± 44.7	188.6 ± 40
Proteins (g)	73.8 ± 15.9	71.6 ± 16	82.7 ± 12.5	80.8 ± 19.6
Fat (g)	77.6 ± 23	67 ± 11.2	68.9 ± 16.7	68.2 ± 30.1
Cholesterol (mg)	227.5 ± 76.3	238.7 ± 109.2	249.7 ± 90.6	231.8 ± 86.1
Fiber (g)	12.4 ± 6	9.5 ± 3.5	14.6 ± 7.5	11.5 ± 5.6

C: Control group; C + C: Control group supplemented with Cranberries; M: Mastitis group; M + C: Mastitis group supplemented with Cranberries. BMI: Body Mass Index. The results are expressed as mean ± standard deviation.

## Data Availability

Data is contained within the article.
